# Development of an Online and Offline Integration Hypothesis for Healthy Internet Use: Theory and Preliminary Evidence

**DOI:** 10.3389/fpsyg.2018.00492

**Published:** 2018-04-13

**Authors:** Xiaoyan Lin, Wenliang Su, Marc N. Potenza

**Affiliations:** ^1^Department of Applied Psychology, School of Humanities and Social Sciences, Fuzhou University, Fuzhou, China; ^2^Department of Sociology, Peking University, Beijing, China; ^3^Institute of Psychological and Cognitive Sciences, Fuzhou University, Fuzhou, China; ^4^Department of Psychiatry, Child Study Center, Department of Neuroscience, and the National Center on Addiction and Substance Abuse, Yale School of Medicine, New Haven, CT, United States; ^5^Connecticut Mental Health Center, New Haven, CT, United States

**Keywords:** integration hypothesis, integration principles, rich get richer, social compensation, Internet addiction, problematic Internet use, healthy Internet use, Online and Offline Integration Scale

## Abstract

The Internet has become an integral part of our daily life, and how to make the best use of the Internet is important to both individuals and the society. Based on previous studies, an Online and Offline Integration Hypothesis is proposed to suggest a framework for considering harmonious and balanced Internet use. The Integration Hypothesis proposes that healthier patterns of Internet usage may be achieved through harmonious integration of people’s online and offline worlds. An online/offline integration is proposed to unite self-identity, interpersonal relationships, and social functioning with both cognitive and behavioral aspects by following the principles of communication, transfer, consistency, and “offline-first” priorities. To begin to test the hypothesis regarding the relationship between integration level and psychological outcomes, data for the present study were collected from 626 undergraduate students (41.5% males). Participants completed scales for online and offline integration, Internet addiction, pros and cons of Internet use, loneliness, extraversion, and life satisfaction. The findings revealed that subjects with higher level of online/offline integration have higher life satisfaction, greater extraversion, and more positive perceptions of the Internet and less loneliness, lower Internet addiction, and fewer negative perceptions of the Internet. Integration mediates the link between extraversion and psychological outcomes, and it may be the mechanism underlying the difference between the “rich get richer” and social compensation hypotheses. The implications of the online and offline integration hypothesis are discussed.

“…*the paradox of any technological revolution is that you need to go offline in order to find wisdom and emotional clarity to make the best use of your online life.*”

—Pico Iyer

## Introduction

Whether the Internet has a positive or negative impact on individuals has been a controversial issue since its emergence. The Internet has played an increasingly important role in people’s lives, and the border between the Internet and real life has become blurred; however, there is growing concern amongst some regarding problems it may have generated or promoted and the lack of clear guidelines for healthy Internet usage (Yau and Potenza, 2014; Suler, 2016; Anioke, 2017). Previous studies have produced several hypotheses (described below) to help explain the relationship between the online and offline worlds (Kraut et al., 2002; Ellison et al., 2007; Müller et al., 2016).

### Rich Get Richer Hypothesis

The Rich Get Richer Hypothesis (2002) proposes that individuals with higher extraversion or who are more comfortable in social situations would be more likely to use the Internet to extend their social networks and enhance the quality of their friendships (Kraut et al., 2002; Peter et al., 2005). According to this hypothesis, individuals who are extraverted and already have strong social skills would do better in sharing their joys and asking for help online, thereby attaining additional social support and higher life satisfaction through cyberspace (Lei and Liu, 2005; Selfhout et al., 2009; Desjarlais and Willoughby, 2010; Khan et al., 2016). Furthermore, players of Internet games who report greater success in the real world are more likely to play games such as World of Warcraft (WOW) in a healthy manner than those who perceived real-life failings (Snodgrass et al., 2011a). Conversely, the “poor get poorer” according to this hypothesis. People who are introverted, have higher levels of social anxiety, and have poorer social skills and confidence would be more likely to use the Internet to escape from and avoid problems in real life, and this could lead to negative outcomes (Armstrong et al., 2000).

### Social Compensation Hypothesis

On the contrary, the Social Compensation Hypothesis (Poor Get Richer Hypothesis) proposes that individuals with higher levels of social anxiety or lower levels of social support who use the Internet would demonstrate greater well-being than those who also have high social anxiety but do not use the Internet (Gross et al., 2002; Peris et al., 2002; Peter et al., 2005, 2006). According to this hypothesis, the anonymity of the Internet provides individuals with a more comfortable social situation due to a perceived lower risk for self-disclosure because of the lack of nonverbal cues (Schouten et al., 2007). Furthermore, the Internet may provide more opportunities for some people to get social support, explore their self-identities and social identities (Peter et al., 2005), and improve their social skills (Shepherd and Edelmann, 2005), as well as a greater opportunity to utilize online coping resources (van Ingen and Wright, 2016). Additionally, Ellison et al. (2007) proposed that online activities were beneficial for individuals to form weak ties in social networking, which would be very useful for those with lower self-esteem to improve their social capital but would be harmful for those with higher self-esteem since it would reduce their opportunities to maintain their strong offline ties. In other words, the “poor get richer” and the “rich get poorer.”

According to the above hypotheses, Internet use may have positive or negative effects depending on individual differences. As both of the above hypotheses have some supporting evidence, it is important to understand the factors involved in determining when the “rich get richer,” “poor get poorer,” “poor get richer,” and “rich get poorer.”

### An Online and Offline Integration Perspective

The concept of online and offline integration was first proposed by Suler (2000). In his opinion, integration creates synergy, and integrating online and offline living would lead to enriched development and prosperity. He also outlined six integration strategies about how to connect online and offline living (e.g., “telling online companions about one’s offline life,” and “bringing online behavior offline”). The integration perspective emphasizes the harmony and balance between one’s online and offline worlds; that is, living in a bigger integrated world would be better than living in two isolated worlds.

However, the integration perspective is far from well recognized by the academic community and warrants additional theoretical consideration, particularly with respect to promoting healthy patterns of Internet usage. Therefore, the present manuscript aims to advance an Online and Offline Integration Hypothesis that may guide the integration of the cyber and real worlds and promote healthy patterns of Internet use.

## Constructing an Online and Offline Integration Hypothesis

### Why Should Online/Offline Domains Be Integrated? Theoretical Background

System theory focuses on the arrangement and relations between the parts and how they may work together as a whole (Bertalanffy, 1969). One of the important insights from system theory is the *holistic view* on the online/offline relationship. The general principle of holism was summarized long ago by Aristotle in the statement that, “the whole is more than the sum of its parts.” However, it’s clear that the whole can be more than the sum of its parts or less, depending on the way the parts are organized and interact. In a holistic worldview, the world is seen as an integrated whole rather than a dissociated collection of parts (Capra, 1997); therefore, the online and offline worlds should be treated as an integrated whole. If we fail to acknowledge their links and focus exclusively on one of them, undesirable consequences may be encountered.

The second insight from system theory is the importance of establishing priority and cooperation of parts within a system. Competition could possibly happen when there are not enough resources available for everything to happen, so that something takes place at the expense of something else (Mobus and Kalton, 2015). The online and offline worlds may be considered under competition to some extent, because both are competing for the investment of people’s time and energy. If prioritization is not clearly established, this kind of resource limitation may give rise to destructive competitive dynamics (Mobus and Kalton, 2015). Dysfunctional competition may generate poor outcomes, such as conflicts and failures observed in relation to Internet addiction (Yau and Potenza, 2014). In the system of online/offline worlds, it is important that the offline life should take higher priority when competing for personal resources, which means that we should attend more to the demands of our real life. As an alternative to competition, the online and offline worlds could work cooperatively for shared goals. The online world could act like a catalyst to enhance and amplify people’s real life. The system with cooperative functioning would likely have more advantages when competing with systems with internal competition (Mobus and Kalton, 2015). Although cooperation may not yield maximal pay-offs for individual parts, mutual cooperation may result in the best pay-off for the whole system (Majolo et al., 2006; Pothos et al., 2011), generating future benefits (Hauser et al., 2014). Therefore, interactive cooperative dynamics among the online and offline worlds may also promote personal development and adaptation in the long run.

In conclusion, according to system theory, an integration approach may represent an ideal way for the organization of online and offline worlds, which is expected to generate the most advantages for optimal functioning in the current digital environment.

### Overview of the Online and Offline Integration Hypothesis

We propose an Online and Offline Integration Hypothesis, which suggests that a healthier pattern of Internet usage may be achieved through the harmonious integration of people’s online and offline worlds into one complete world, by way of integrating online and offline self-identities, interpersonal relationships, and social functioning in the cognitive and behavioral domains.

**FIGURE 1:**
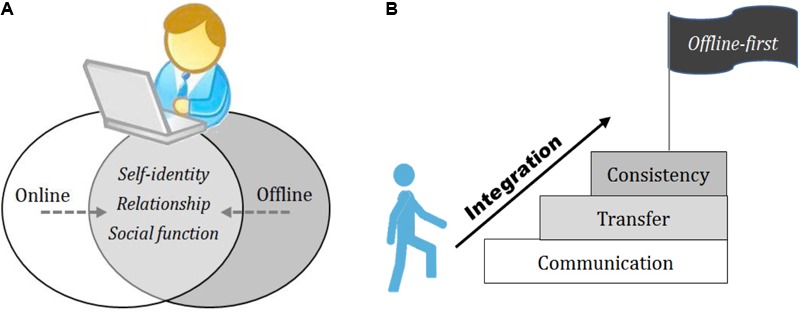
Representative schematic diagram of online/offline integration. **(A)** Integration domains; **(B)** Integration Principles.

Although the cyber world and the real world differ, we propose that they should be combined harmoniously into one world (see **Figure 1A**). The hypothesis proposes that a higher level of harmonious integration may reflect a healthier pattern of Internet usage and lead to better psychological health and well-being. Attempts to avoid real-world experiences or disengage the real world from the online world may generate mental health and social adaptation problems.

### What to Integrate: Three Domains for Online/Offline Integration

Although the six online and offline integration strategies suggested by Suler (2000) have presented valuable insight about how to preserve the harmony and union of the cyber world and the real world, they were mostly focused on the domain of relationships and related behaviors. The importance of self-identity and social function integration has also been described (Weiser, 2001; Bessière et al., 2007; Li et al., 2011; Utz, 2015). In view of the previous literature and theoretical background, we suggest that to promote integration, one should focus on the two worlds’ self-identities, interpersonal relationships, and social function integration in cognitive and behavioral realms.

#### Self-Identity Integration

Self-identity integration emphasizes the balance of self-appraisal in cognition and maintaining consistency in self-presentation of behavior within online and offline worlds. People should demonstrate consistency in self-appraisal and self-acceptance between the online and offline worlds, and likewise experience few discrepancies of appraisals from others. They should also present a similar personal image and demonstrate similar behavioral styles in both online and offline domains.

Studies have provided some evidence to support this concept. For example, studies suggest that online–offline self-discrepancy (Bessière et al., 2007) or actual–ideal self-discrepancy and escapism (Li et al., 2011) may lead to lower psychological well-being and excessive involvement in Internet activities. For instance, Internet gamers who have maladaptive cognitions related to the cyber world are more likely to have higher Internet gaming disorder symptomatology (King and Delfabbro, 2016). By contrast, individuals who are better able to express and disclose their true selves over the Internet have been found to be more likely to have made intimate online friends and to have moved these friends into the real world (McKenna et al., 2002; Valkenburg and Peter, 2007b; Utz, 2015).

#### Interpersonal Relationship Integration

Relationship integration involves online communication as a supplement to face-to-face real-world relationships and a preference for online communication with known and non-anonymous friends versus unknown individuals. People should transfer relationships through the two worlds via online communications with known (versus unknown) persons and meetings with online friends in real life to achieve a greater overlap of the two groups. They might also let offline friends know what is happening in their online lives and vice versa.

Online/offline relationship integration can produce better outcomes. For example, communicating with existing friends online may result in higher friendship quality and increased well-being, but chatting with strangers may not show this effect (Valkenburg and Peter, 2007a, 2009; van den Eijnden et al., 2008). Playing online games with known real-life friends may help players avoid problematic Internet use and improve their offline lives through their online success and achievement (Snodgrass et al., 2011b). These players may also experience less loneliness in the online world than players not playing with known people (Martonèik and Lokša, 2016). Eklund (2015) explained that relationships are hard to maintain through the online world alone, unless there are further connections such as offline ties and other similarities.

#### Social Function Integration

Social functioning involves an individual’s interactions with their environments and their fulfillment of roles within the environments (Weissman, 1975; Bosc, 2000). Social function integration suggests a motivation for Internet use is to serve real-life functions (e.g., social, school, work, or family activities), and avoiding seeing cyberspace as an escape from real-life problems. From a behavioral perspective, online activities should largely relate to academic/job/daily life functioning and are accepted by others around the individual (e.g., family members) as promoting real-life functioning.

The Internet’s social and psychological effects depend upon the functions it serves for users (Weiser, 2001). A practical or utilitarian orientation of Internet use could have a beneficial effect on psychological well-being by improving social integration (Weiser, 2001). For example, studies have demonstrated that heavier recreational Internet use was related to poorer academic performance (Kubey et al., 2001), but academic use of the Internet could improve school performance (Kim et al., 2017). Additionally, the influence of social functions on academic and occupational activities has become an important consideration for problematic Internet use (Young, 1998; Chen et al., 2003; Lei and Yang, 2007), with findings indicating the importance of using the Internet to serve real-life functioning rather to escape from it.

### How to Integrate: Four Principles of Online/Offline Integration

We propose four general principles of online/offline integration— **C**ommunication, **T**ransfer, **C**onsistency, and **O**ffline first (CTCO) Principles. The CTCO principles are proposed to be the major approaches for achieving online/offline integration (see **Figure 1B**).

#### Communication Principle

Communication represents one important factor with respect to intersystem relationships (Kuhn, 1974). For the Integration Hypothesis, this means that the online and offline domains should not be separated into two isolated worlds, but they should be bridged through exchanges of information. According to the communication principle, people are advised to introduce their online world (e.g., feelings, activities, and friends) to their offline world, and vice versa. Being able to exchange information freely and openly between the two worlds is a first step to achieving integration.

Communication helps to enhance mutual understanding across the online and offline worlds, thus minimizing differences, facilitating mutual learning, and promoting coordination to function as a whole. Communication may also help people to establish healthier patterns of Internet usage. Not having secretive patterns of Internet use may promote healthy usage and prevent problematic use.

#### Transfer Principle

Based on communication between the two worlds, people can further achieve integration via transference. The transfer principle encompasses the notion that one world (e.g., online) could be the new source of development for another world (e.g., offline), and they could learn from each other. Due to the different features of the online and offline worlds, they may provide more space and possibilities for the person to experiment with new identities, explore new abilities, and get acquainted with new friends. When developing or extending from one world into another, individuals may transfer these new ideas, concepts, or information. By practicing the transfer principle, the boundaries between the worlds may be weakened and their coordination promoted.

#### Consistency Principle

Although the features of the online and offline worlds are different, it is important for a harmonious union for there to be consistency between them. Such consistency may involve similarities in identities presented, equivalent appraisals, and complementary goals, amongst other factors. The greater the similarities presented in the two worlds, the more likely a complete and consistent whole may be achieved. It should be noted that consistency is not a static state, but rather a dynamic process from discrepancy to consistency achieved through effective communication and transfer.

#### Offline First Principle

Integration does not mean that the online and offline worlds are parallel and equal. As human beings, we operate in the physical world, and nobody can survive solely in a digital world. Furthermore, we have adapted to the physical world for millions of years through evolution, whereas a cyber world has only been in existence for a few decades. Relatedly, people who overly detach from the real world may be susceptible to physical and mental disorders. In this sense, online behaviors should serve people’s real lives and be mostly integrated into the basis of real life, rather than the other way around. To establish this kind of priority is also necessary when online/offline domains compete in the resource-limited life of a human being (Mobus and Kalton, 2015).

## Examining the Hypothesis

As suggested by our hypothesis that higher online and offline integration level of Internet use would lead to better psychological outcomes, we hypothesized that greater integration would be associated with less Internet addiction, more pros, and fewer cons of Internet use, less loneliness, and greater life satisfaction among college students in this study (H1). In previous studies, extraverted individuals benefited more and had better psychological outcomes than introverted individuals from Internet use (Kraut et al., 2002; Muller et al., 2014). We hypothesized that extraversion would correlate with a higher level of integration (H2), and the level of integration would mediate the relationship between extraversion and those psychological measures (e.g., Internet addiction, loneliness, and life satisfaction; H3). Since the “rich get richer” hypothesis and social compensation hypothesis have conflicts in predicting whether extraverted and introverted individuals would benefit or get worse from Internet use, we hypothesized that integration should be taken into consideration in this phenomenon, and assumed that both extraverted and introverted individuals could “get richer” (have better psychological correlates) under higher integration levels than those low in integration (“get poorer”; H4).

### Method

#### Participants

This research was approved by the Research Ethics Committee at the Institute of Psychological and Cognitive Sciences, Fuzhou University. All of the participants were college students recruited from Fujian Jiangxia University and Fujian Agriculture and Forestry University, located in the southeast of China. They volunteered to answer the questionnaires anonymously through an online survey and a total of 742 respondents completed the questionnaires. After screening out individuals providing inappropriate or invalid responses (*n* = 116), we obtained 626 valid responses for further analysis. Of the final sample, 260 (41.5%) were male, and the sample had a mean age of 20.1 (*SD* = 1.4).

#### Measures

##### Online and offline integration scale (OOIS)

A self-developed, 15-item OOIS questionnaire was used to assess participants’ levels of online and offline integration (see Appendix 1 in Supplementary Material). According to the framework of the online/offline integration hypothesis, the OOIS has three subscales, each having five items, reflecting self-identity integration (SI, Cronbach α = 0.69), relationship integration (RI, Cronbach α = 0.66), and social function integration (SFI, Cronbach α = 0.57). The scale showed a good factor model fit (χ^2^ = 386.95, χ^2^/*df* = 4.45, *RMSEA* = 0.075, *GFI* = 0.92, *CFI* = 0.89). Each item asks about the integration of online and offline experiences (e.g., “My online friends know well how I am in real life”). Participants responded to the items using a 4-point Likert scale, where 1 = strongly disagree; 2 = disagree; 3 = agree; and 4 = strongly agree. The reliability coefficient of the total scale was 0.75 in the study. The OOIS score was calculated as the sum of the three subscales score, and a higher OOIS score indicated a higher level of integration.

##### Internet use decisional balance questionnaire (IDBQ)

The IDBQ is based on the Transtheoretical Model (Prochaska et al., 1992) and is designed to measure people’s decisional balance regarding their Internet usage (Liu et al., 2010). The questionnaire has 38 items, including pros and cons subscales. The pros subscale is composed of 16 items (e.g., “the Internet relieves the tension of study or life.”), while the cons subscale has 22 items (e.g., “the Internet made me fail to finish my academic homework on schedule.”). The IDBQ showed good reliability and validity and could serve as a measurement tool of Chinese university students’ decisional balances regarding their Internet use (Liu et al., 2010). Participants respond to the items using a 4-point Likert scale (1 = strongly disagree, 4 = strongly agree). The reliability coefficient in the study was 0.91 for the pros subscale and 0.94 for the cons subscale.

##### Internet addiction diagnostic questionnaire (IADQ)

The IADQ is an 8-item questionnaire developed by Young (1998) to screen for Internet addiction. Answers of “Yes” score 1; answers of “No” score 0. In this study, the Cronbach’s α was 0.73.

##### Satisfaction with life scale (SWLS)

The SWLS is a short 5-item instrument designed to measure global subjective feelings of satisfaction with one’s life (Diener et al., 1985). Participants respond to items using a 4-point Likert scale (1 = strongly disagree, 5 = strongly agree). The Cronbach’s α in this study was 0.87, indicating that the scale demonstrated high internal consistency.

##### UCLA loneliness scale

A 20-item questionnaire was used to measure subjective social loneliness (Russell, 1996). Participants respond to the items using a 4-point scale (1 = never, 2 = rarely, 3 = sometimes, 4 = usually). The coefficient alpha in this study was 0.83.

##### Extraversion

Extraversion was extracted from the brief version of Chinese Big Five Personality Inventory (CBF-PI-B; Wang et al., 2011). The CBF-PI-B is a 40-item scale consisting of five subscales: agreeableness, openness, extraversion, neuroticism, and conscientiousness. Scale items are rated on a 6-point Likert scale (1 = disagree strongly, 6 = agree strongly). Support for the CBF-PI-B’s validity has been demonstrated by its relationship to the Big Five Inventory (*r* = 0.58∼0.83, Wang et al., 2011). The extraversion subscale has eight items, and its Cronbach’s α for the present study was 0.82, which indicated good internal consistency.

#### Statistical Analyses

All statistical analyses were conducted using SPSS (version 19, IBM Corp.) Pearson correlations were used to access bivariate associations. A hierarchical multiple regression was employed to examine the relationship between extraversion, integration, and psychological outcomes.

Mediation effects were tested with the SPSS macros PROCESS (v3.0) for bootstrapping as provided by Hayes (2017). Indirect mediating effects were evaluated with 95% confidence intervals using the percentile method based on 5,000 bootstrap samples. If the confidence interval does not contain zero, then it indicates that the indirect effect can be considered statistically significant (Hayes, 2017).

Based on the mean score of the OOIS, the participants were divided into high-integration (larger than mean, *n* = 262) and low-integration (less than mean, *n* = 364) groups. Similarly, the participants were divided into extraverted (*n* = 326) and introverted (*n* = 300) groups based on the scores that were above or below the mean extraversion score. Then, 2 × 2 ANOVAs were performed with extraversion (extravert and introvert) and integration (low and high) serving as between-subject variables. Separate analyses were performed for Internet addiction, loneliness, and life satisfaction. To more easily compare the results, *z* scores for the dependent variables were used. Partial *η*^2^ was given as effect size when appropriate. Bonferroni correction was used to adjust for the results of multiple comparisons in simple effects.

### Results

#### Descriptive Statistics and Correlations

Descriptive statistics of and correlations between study variables are depicted in **Table 1**. The three OOIS subscales were positively correlated with each other (*r* = 0.20 to 0.38, *ps* < 0.01). As hypothesized in H1, SI, RI, SFI, as well as the total score of OOIS were negatively correlated with Internet addiction (*r* = -0.15 to -0.34, *ps* < 0.01), cons (*r* = -0.12 to -0.36, *ps* < 0.01) and loneliness (*r* = -0.27 to -0.43, *ps* < 0.01). RI, SF, and OOIS positively correlated with pros (*r* = 0.10∼0.15, *ps* < 0.01), and OOIS was not correlated with SI (*r* = 0.01, *ns*). OOIS and its three subscales were also positively correlated with life satisfaction (*r* = 0.13–0.23, *ps* < 0.01). As predicted in H2, extraversion was found positively correlated with OOIS subscales and its total scores (*r* = 0.20–0.31, *ps* < 0.01).

**Table 1:** Descriptive statistics of and zero-order correlations between study variables.

	1	2	3	4	5	6	7	8	9	10	11	12	13
(1) Age	1												
(2) Gender^a^	0.12**	1											
(3) SI	0.01	-0.08*	1										
(4) RI	0.06	-0.19**	0.38**	1									
(5) SFI	-0.06	-0.01	0.21**	0.20**	1								
(6) OOIS	0.01	-0.14**	0.76**	0.74**	0.63**	1							
(7) Internet time^b^	0.15**	-0.06	-0.06	-0.03	-0.13**	-0.10*	1						
(8) Internet Addiction	0.10*	-0.12**	-0.26**	-0.15**	-0.33**	-0.34**	0.17**	1					
(9) Pros	0.01	-0.02	0.01	0.15**	0.10**	0.12**	0.13**	0.15**	1				
(10) Cons	0.08	0.03	-0.22**	-0.12**	-0.36**	-0.32**	0.20**	0.49**	0.29**	1			
(11) Extraversion	0.06	0.11**	0.20**	0.24**	0.22**	0.31**	-0.04	-0.19**	0.09*	-0.13**	1		
(12) Loneliness	0.03	0.06	-0.36**	-0.30**	-0.27**	-0.43**	0.02	0.34**	-0.08*	0.41**	-0.41**	1	
(13) Life satisfaction	-0.02	0.04	0.13**	0.16**	0.22**	0.23**	0.01	-0.24**	0.09*	-0.18**	0.23**	-0.38**	1
*M*	20.07	/	15.31	14.00	13.79	43.11	5.45	2.25	46.50	44.24	28.95	44.47	14.49
*SD*	1.36	/	2.21	2.07	1.95	4.47	3.15	1.94	10.55	14.62	6.10	8.21	3.80

*SI, Self-identity Integration; RI, Relationship Integration; SFI, Social Function Integration; OOIS, total score of Online and Offline Integration Scale. ^*a*^Gender was coded as male = 1, female = 0. ^*b*^Internet time was measured as the number of online hours per day. ^∗^*p* < 0.05, ^∗∗^*p* < 0.01.*

#### Does Integration Mediate the Relationship Between Extraversion and Psychological Outcomes?

**FIGURE 2:**
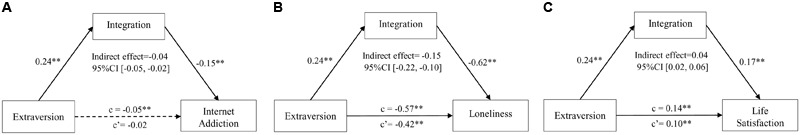
Integration mediates relationships between extraversion and psychological outcomes (*N* = 5000 bootstrapping resamples). Dependent psychological outcome variables: **(A)** Internet addiction; **(B)** loneliness; **(C)** life satisfaction. Integration was measured as the total score of Online and Offline Integration Scale. All paths are quantified with unstandardized regression coefficients. ^∗^*p* < 0.05, ^∗∗^*p* < 0.01. Path *c* = total (non-mediated) effect; Path *c*’ = direct (controlling mediator) effect.

To test the hypothesized mediating effect of integration (H3), the indirect and direct effects of extraversion on psychological outcomes were calculated with 5,000 bootstrap samples. Age, gender, and Internet time were included as covariate variables. The bootstrap results showed that integration fully mediated the relationship between extraversion and Internet addiction, and the estimate of mediation effect was -0.04 with a 95% bootstrap CI of -0.05 to -0.02 (see **Figure 2A**). The mediation effect on loneliness was significant and partial, and the estimate was -0.15 with a 95% bootstrap CI of -0.22 to -0.10 (see **Figure 2B**). The mediation effect on life satisfaction was also significant and partial, and the estimate was 0.04 with a 95% bootstrap CI of 0.02–0.06 (see **Figure 2C**). These results indicated that H3 was supported. We also conducted a series of hierarchical multiple regression models on those three psychological outcomes. Age, gender, and Internet time were entered at the first step, and then extraversion at step 2, and finally the three OOIS subscales SI, RI, and SFI were entered at step 3. The results are shown in Supplementary Table S1.

#### Differences in Relationships Between Psychological Measures, Extraversion, and Integration

To examine the H4, two-way ANOVAs were conducted to examine the statistical effects of extraversion (extravert and introvert) and integration (low and high) on Internet addiction, loneliness, and life satisfaction separately.

**FIGURE 3:**
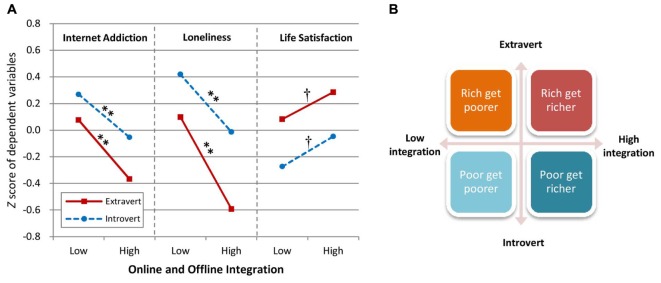
Integration, extraversion, and their psychological correlates. **(A)** Mean Z score of Internet addiction, loneliness, and life satisfaction as a function of online/offline integration (low or high) and extraversion (extraverted or introverted). **(B)** Diagram of the psychological effects of different online and offline integration levels for extraverts and introverts. ^†^*p* < 0.1, *^∗^p* < 0.05, *^∗∗^p* < 0.01.

For Internet addiction, results indicated a significant main effect for integration, *F*(1,622) = 22.12, *p* < 0.01, partial *η*^2^ = 0.034, and also for extraversion, *F*(1,622) = 9.12, *p* < 0.01, partial *η*^2^ = 0.015. Overall, the high integration group reported a significantly lower proportion of Internet addiction (*M* = -0.26, *SD* = 0.86) than the low integration group (*M* = 0.19, *SD* = 1.05). The extraverted group also reported a significantly lower tendency to Internet addiction (*M* = -0.16, *SD* = 0.92) than to the introverted group (*M* = 0.17, *SD* = 1.06). The extraversion × integration interaction was not statistically significant, *F*(1,622) = 0.55, *ns*, partial *η*^2^ = 0.001. Simple effects analyses indicated that as compared to low integration, high integration in both the extraverted and introverted groups demonstrated a lower proportion of Internet addiction (*ps* < 0.01). Relevant means and comparisons are presented in **Figure 3A**.

For loneliness, results indicated a significant main effect for integration, *F*(1,622) = 53.12, *p* < 0.01, partial *η*^2^ = 0.079, and also for extraversion, *F*(1,622) = 37.22, *p* < 0.01, partial *η*^2^ = 0.056. Overall, the high integration group reported a significantly lower level of loneliness (*M* = -0.40, *SD* = 1.06) as compared to the low integration group (*M* = 0.28, *SD* = 0.84). The extraverted group also reported a significantly lower tendency to loneliness (*M* = -0.28, *SD* = 1.01) as compared to the introverted group (*M* = 0.30, *SD* = 0.90). The extraversion × integration interaction was not statistically significant, *F*(1,622) = 2.81, *ns*, partial *η*^2^ = 0.005. Simple effects analyses indicated that compared to low integration, high integration in both the extraverted and introverted groups demonstrated significantly less loneliness (*ps* < 0.01). Relevant means and comparisons are presented in **Figure 3A**.

For life satisfaction, results indicated a significant main effect for integration, *F*(1,622) = 6.85, *p* < 0.01, partial *η*^2^ = 0.011, and also for extraversion, *F*(1,622) = 17.45, *p* < 0.01, partial *η*^2^ = 0.027. Overall, the high integration group reported a significantly higher level of life satisfaction (*M* = 0.17, *SD* = 1.02) than the low integration group (*M* = -0.12, *SD* = 0.96). The extraverted group also reported a significantly higher proportion of life satisfaction (*M* = 0.19, *SD* = 0.99) than the introverted group (*M* = -0.21, *SD* = 0.97). The extraversion × integration interaction was not statistically significant, *F*(1,622) = 0.02, *ns*, partial *η*^2^ < 0.001. Simple effects analyses indicated that compared to low integration, high integration in both the extraverted and introverted groups demonstrated a marginally significant higher level of life satisfaction (*p* = 0.062 for extravert and *p* = 0.067 for introvert). Relevant means and comparisons are presented in **Figure 3A**.

The above results indicate that extravert has better psychological correlates (“rich”) than introvert (“poor”) in general. However, as shown in **Figure 3A**, extraverted individuals with high integration will have better psychological measures (“rich get richer”) than those low in integration (“rich get poorer”). Similarly, introverted individuals with high integration of Internet use will have better psychological measures (“poor get richer”), than those low in integration (“poor get poorer”). Therefore, H4 was supported. A diagram of the psychological effects of different online/offline integration levels for extraverted and introverted groups is presented in **Figure 3B**.

## General Discussion

The goal of the study was to try to introduce and further develop a new theoretical perspective on cyberpsychology based on Suler (2000)’s earlier work, namely the Online and Offline Integration Hypothesis. The hypothesis is in line with System Theory on how to organize the relationship of online and offline worlds in a cooperative and productive way (Mobus and Kalton, 2015). CTCO principles are proposed as the major approaches for achieving online/offline integration, in which communication and transfer principles help to weaken boundaries between the online/offline worlds and promote their coordination, while the consistency and offline-first principles may provide direction to the integration process. Based on previous findings, the hypothesis also assumes that the self-identity, interpersonal relationship, and social functioning are crucial domains that people should prioritize with respect to integration. The hypothesis emphasizes the importance of creating synergy between the online and offline worlds, suggesting that a healthy cyber world does not extend or replace the real world. Instead, individuals need an integration process for both and should demonstrate a balance between online and offline experiences.

The hypothesis proposes that better integrated Internet use is advantageous. Consistent with our conceptual framework, the present study demonstrated that online/offline integration was positively correlated with life satisfaction and positive perceptions of the Internet (pros), as well as negatively correlated with measures of Internet addiction, loneliness, and negative perceptions of the Internet (cons). Some personal characteristics may provide strengths for an integrative approach and therefore make the individual more likely to be “richer.” For example, we found that people who had higher levels of extraversion were more likely to have higher levels of online/offline integration (*r* = 0.31, *p* < 0.01), and integration mediated the relationships between extraversion and psychological measures. This result may partly explain the phenomenon of the “rich get richer” in the Kraut et al. (2002) study, from which using the Internet predicted better outcomes for those more extraverted but worse outcomes for more introverted individuals.

Our study may also help explain apparent controversies between several competing hypotheses including the “rich get richer” hypothesis (Kraut et al., 2002) and social compensation hypothesis (“poor get richer”; Gross et al., 2002; Peter et al., 2005). As shown in **Figure 3**, introverted individuals may benefit from high integration of Internet use (poor get richer), and extraverted individuals may get worse from low integration (rich get poorer), consistent with the social compensation hypothesis. On the other hand, introverted individuals may get worse from low integration (poor get poorer), and extraverted individuals may benefit from high integration (rich get richer), consistent with the “rich get richer” hypothesis. Hence, integration may be the mechanism underlining the difference in predictions from the social compensation hypothesis and the “rich get richer” hypothesis. That is, the “rich” (e.g., extraverted group) or “poor” (e.g., introverted group) may not necessarily get richer or poorer *per se*, with integration level contributing to the direction. More research is needed to examine how online and offline integration may relate to psychological variables, particularly over time as might be examined in longitudinal studies.

### Potential Applications of the Integration Hypothesis

The Integration Hypothesis has important implications. It may be possible to help prevent Internet addiction by improving the integration levels of Internet-use behaviors. Individuals with problematic Internet use may have difficulties maintaining balance or controlling their Internet use in relation to everyday life (Yau and Potenza, 2014). Such individuals may have maladaptive cognitions with respect to the two worlds, and they may use the Internet to escape from difficulties in the real world (Greenfield, 1999). They may also neglect important relationships (Young, 1998) and encounter problems in the workplace (Griffiths, 2010) or at school (Akhter, 2013). Although multiple intervention programs for Internet addiction have been developed and tested to varying degrees (King et al., 2011), the Integration Hypothesis has potential value in bringing in new ideas for clinical or educational interventions for this population. For example, the hypothesis emphasizes the importance of self-identity, relationships, and social function integration for healthy Internet use, and our study had provided initial data that showed that high levels of integration in these three domains correlates with lower levels of Internet addiction. Interventions may focus on those domains and promote online/offline integration with CTCO principles in practice. The integration should make offline-first as the orientation, and may facilitate integration level through communication as a first step, with subsequent work involving transference of each domain to the other to achieve more consistency and harmony between the online and the real world. Since the Internet addicts usually use the Internet as an escape (Young and Brand, 2017), programs may be developed to decrease people’s problematic Internet use by improving the level of integration of online and offline spaces, and such possibilities should be explored and examined directly.

The hypothesis is not only a theoretical framework to evaluate how people use the Internet but also a powerful tool to estimate the potential influence of the cyber environment through integration strategies. A first strategy may be related to immersion: the greater an immersion in a digital product, the greater the tendency people may have to avoid the real world (Snodgrass et al., 2011b); thus, they may experience a split between the digital and real-world environments. For example, Augmented Reality (AR), which blends cyberspace into the real world, may promote online/offline integration (Suler, 2016, p. 85), whereas Virtual Reality (VR), which is an immersive, interactive experience generated by a computer, may promote a dissociation from the real world. Thus, the latter may be more likely to lead to non-integration and problematic use, although this possibility warrants direct empirical examination. A second strategy may involve people whom individuals have contact with and whether they are known or unknown in real life, as well as whether identified or anonymous accounts are encouraged. Mobile applications like LinkedIn and WhatsApp, which were primarily designed for people to contact and share with other persons they already know (e.g., friends and family), can be labeled as a higher integration communication tool than those stranger/anonymity-orientated social apps like GaGa or Yik Yak. Data suggest that playing with known people in an online game may generate lower perceived loneliness than playing with unknown people (Martonèik and Lokša, 2016). A third strategy may involve social networking products and communication cues. Photo, voice, and video interactions are prominent in apps like Instagram or Skype, which utilize a large amount of visual or auditory information more typical of traditional face-to-face interactions and are theoretically more integrated than those that are mainly text-based social networking service (SNS), like Facebook and Twitter. Compared with typing, visual and auditory cues used in interaction could form a higher quality of communication, develop better friendships, and reduce perceived loneliness (Martonèik and Lokša, 2016). In addition to the above, there are other possible strategies that could be derived from the integration principles. This study suggests that developers should consider integration strategies when designing a product, especially if they aim to strike a balance between entertainment and connectivity with real life. Different strategies employed by generators on the products they are developing may result in people adopting different online/offline integration levels.

### Limitations and Future Research

Although the present study takes an initial step in constructing the core concepts of the Integration Hypothesis and provide preliminary evidence that different levels of integration may have different psychological outcomes, there are limitations that should be addressed. First, although the integration domains and principles proposed here were based on previous literatures and the System Theory, they still need to be more carefully discussed and examined in the future. Second, the OOIS was developed and examined based on college students in China, and future studies should examine its validity in other age groups and in other cultures. Third, the structure of the current scale was based on domains rather than the principles. That being said, the integration principles are reflected among the OOIS items. For example, the item, “My offline friends or my family members know well how I am on the Internet,” reflects the principle relating to communication. Similarly, the item, “People with whom I communicate on the Internet and with whom I communicate in real life are mostly the same,” reflects the principle relating to consistency. Nonetheless, future studies should measure the principles directly to evaluate how individuals approach integration. Finally, the results of the present study were based on a correlational design, so we could not identify a cause-and-effect relationship between the online/offline integration and the outcome measures; future studies may utilize longitudinal methods or experimental design to investigate possible causal relationships.

Future studies should examine the extent to which online and offline integration levels may account for potential differences in relationships between individuals and Internet-use behaviors, particularly as integration may act as a moderating or mediating variable between specific individual differences and psychological outcomes. In this process, examination of other factors (e.g., potential influences of relative socioeconomic advantage versus disadvantage) should be considered. Moving forward, numerous Internet products may have more direct connections with real life, studies that compare the relationships between different products (or aspects thereof) with different integration tendency features (e.g., anonymity and familiarity, simulated level of social presence, and immersion) would be interesting, valuable, and potentially impactful with respect to public health considerations. From a public health perspective, factors that prospectively relate to better or worse health over time are important to identify. It thus may be very meaningful for researchers to examine which features may predict integration tendencies over time, particularly if integration levels are found to moderate relationships with health and well-being. The study of protective and risk factors as they relate to levels of online/offline integration thus may have important practical and public health implications.

## Conclusion

The study introduced a new theoretical perspective on cyber-psychology, the Integration Hypothesis, which provides a new framework for examining the relationship between online and offline worlds. The hypothesis is proposed to unite self-identity, interpersonal relationships, and social functioning in cognitive and behavioral domains by following the principles of communication, transfer, consistency, and “offline-first” priorities. The study suggests that more harmonious integration of online and offline experiences is associated with less Internet addiction, more pros and fewer cons regarding Internet use, less loneliness, more extraversion, and greater life satisfaction. Integration mediates relationships between extraversion and psychological outcomes, and integration may be a mechanism underlying seemingly different predictions from the “rich get richer” and social compensation hypotheses. The proposed integration hypothesis has a wide range of implications for our understanding of Internet-use behaviors.

## Author Contributions

WS was responsible for the theoretical concept and study design. XL contributed to the data collection and preliminary analysis. WS and XL wrote the first draft of the manuscript. MP provided the critical revision of the manuscript for intellectual content. All authors contributed to and have approved the final manuscript.

## Disclaimer

The views presented in this manuscript represent those of the authors and not necessarily those of the funding agencies who had no input into the content of the manuscript.

## Conflict of Interest Statement

MP has consulted for and advised Shire, INSYS, Rivermend Health, Opiant/Light Lake Therapeutics and Jazz Pharmaceuticals; received research support (to Yale) from the Mohegan Sun Casino and the National Center for Responsible Gaming; participated in surveys, mailings, or telephone consultations related to drug addiction, impulse control disorders, or other health topics; consulted for law offices and gambling entities on issues related to impulse control and addiction; and given academic lectures in grand rounds, CME events, and other clinical/scientific venues. The other authors declare that the research was conducted in the absence of any commercial or financial relationships that could be construed as a potential conflict of interest.
