# Corrigendum: Development of an Online and Offline Integration Hypothesis for Healthy Internet Use: Theory and Preliminary Evidence

**DOI:** 10.3389/fpsyg.2019.00106

**Published:** 2019-01-31

**Authors:** Xiaoyan Lin, Wenliang Su, Marc N. Potenza

**Affiliations:** ^1^Department of Applied Psychology, School of Humanities and Social Sciences, Fuzhou University, Fuzhou, China; ^2^Department of Sociology, Peking University, Beijing, China; ^3^Institute of Psychological and Cognitive Sciences, Fuzhou University, Fuzhou, China; ^4^Department of Psychiatry, Child Study Center, Department of Neuroscience, and the National Center on Addiction and Substance Abuse, Yale School of Medicine, New Haven, CT, United States; ^5^Connecticut Mental Health Center, New Haven, CT, United States

**Keywords:** integration hypothesis, integration principles, rich get richer, social compensation, Internet addiction, problematic Internet use, healthy Internet use, online and offline integration scale

In the original article, there was a mistake in Figure 3A as published. Due to a drawing error, the line style of introvert and extravert in the panel of “Loneliness” was drawn incorrectly. The corrected Figure 3 appears below.

**Figure 3:**
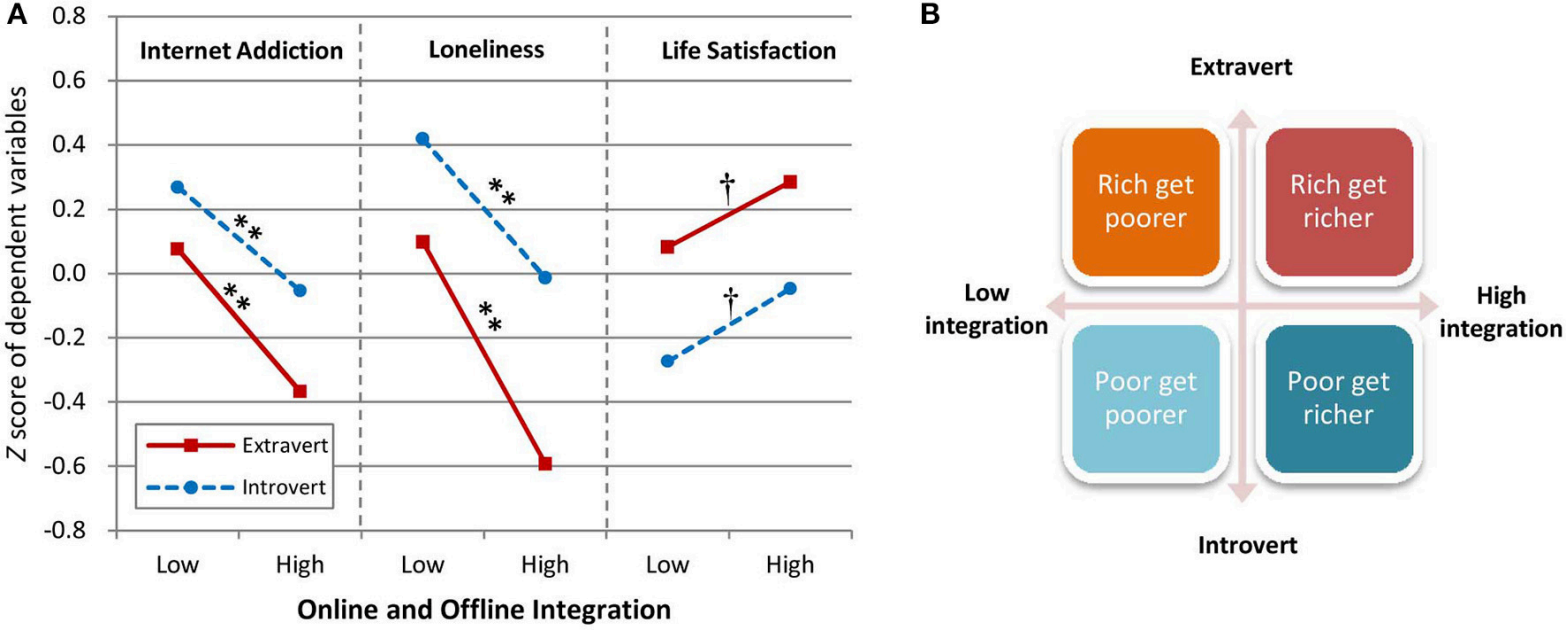
Integration, extraversion, and their psychological correlates. **(A)** Mean Z score of Internet addiction, loneliness, and life satisfaction as a function of online/offline integration (low or high) and extraversion (extraverted or introverted). **(B)** Diagram of the psychological effects of different online and offline integration levels for extraverts and introverts. ^†^*p* < 0.1, ^*^p < 0.05, ^**^p < 0.01.

The authors apologize for this error and state that this does not change the scientific conclusions of the article in any way. The original article has been updated.

## Conflict of Interest Statement

The authors declare that the research was conducted in the absence of any commercial or financial relationships that could be construed as a potential conflict of interest.

