# Supplementary material for: Development of an Online and Offline Integration Hypothesis for Healthy Internet Use: Theory and Preliminary Evidence
Source: Front Psychol. 2018 Apr 13;9:492. doi: 10.3389/fpsyg.2018.00492 (PMC5908967; doi:10.3389/fpsyg.2018.00492)
Supplement: Supplementary file 1 [file Table_1.DOCX]

Supplementary Material

Development of An Online and Offline Integration Hypothesis for Healthy Internet Use: Theory and Preliminary Evidence

Xiaoyan Lin, Wenliang Su*, Marc N. Potenza

*** Correspondence:** Wenliang Su: suwenliang@fzu.edu.cn

# Appendix 1: Online and Offline Integration Scale

The following statements are related to your perceptions and behaviors toward your online and real lives. Please decide whether each statement applies to you using a scale with numbers corresponding to 1=strongly disagree, 2=disagree, 3=agree, and 4=strongly agree.

1. I prefer my online self rather than my offline self. (*R*)
2. My offline friends or my family members know well how I am on the Internet.
3. I believe that killing time is the most important function of the Internet. (*R*)
4. The disposition that I present on the Internet is much different from the one that I present in real life. (*R*)
5. My friends in the Internet know well how I am in the real life.
6. I have benefited much from using the Internet for my study or work.
7. I always like to play roles that differ from my offline roles when I am on the Internet. (*R*)
8. People with whom I communicate on the Internet and with whom I communicate in real life are mostly the same.
9. Most of my online activities serve the needs of my study, job or daily life.
10. Compared to my real-life self, that on the Internet is my ideal self. (*R*)
11. On the Internet, I mostly interact with my offline friends or family members.
12. Most of my Internet time is unrelated to my school work or occupational tasks. (*R*)
13. My online and offline selves look like two completely different people. (*R*)
14. What I post on the Internet is public to my offline friends and family members.
15. I have often been told that my online activities are not related to my responsibilities at work, school or home. (*R*)

*Note*: R= reverses item

*Factor 1: Self-Identity Integration：1, 4, 7, 10, 13*

*Factor 2: Relationship Integration: 2, 5, 8, 11, 14*

*Factor 3: Social Function Integration: 3, 6, 9, 12, 15*

# Appendix 2: Hierarchical multiple regression analysis results

After controlling for demographic variables (age, gender), Internet time and extraversion, SI and SFI statistically predicted Internet addiction (Step 3). All three subscales of OOIS statistically predicted loneliness, and only RI and SFI statistically predicted life satisfaction. Extraversion statistically predicted Internet addiction in Step 2, but showed no significant relationship in Step 3 when variables of OOIS subscales were entered into the model. Results were shown in Table S1.

**TABLE S1** | Hierarchical multiple regression models of demographic, Internet time, extraversion and online/offline integration statistically predicting Internet addiction, loneliness, and life satisfaction.

| Model | Predicting variables | Internet addiction | |  | Loneliness | |  | Life satisfaction | |
| --- | --- | --- | --- | --- | --- | --- | --- | --- | --- |
|  |  | *β* | *t* |  | *β* | *t* |  | *β* | *t* |
| Step 1 | Age | 0.09 | 2.14* |  | 0.02 | 0.46 |  | -0.02 | -0.44 |
|  | Gender ^a^ | -0.11 | -2.82** |  | 0.06 | 1.51 |  | 0.05 | 1.15 |
|  | Internet time ^b^ | 0.15 | 3.64** |  | 0.02 | 0.40 |  | 0.01 | 0.31 |
| Step 2 | Age | 0.10 | 2.45* |  | 0.05 | 1.22 |  | -0.03 | -0.82 |
|  | Gender | -0.10 | -2.45* |  | 0.10 | 2.67** |  | 0.03 | 0.65 |
|  | Internet time | 0.14 | 3.51** |  | -0.00 | -0.02 |  | 0.02 | 0.55 |
|  | *Extraversion* | -0.17 | -4.33** |  | -0.42 | -11.28** |  | 0.23 | 5.82** |
| Step 3 | Age | 0.09 | 2.39* |  | 0.05 | 1.38 |  | -0.03 | -0.78 |
|  | Gender | -0.14 | -3.56** |  | 0.05 | 1.33 |  | 0.06 | 1.40 |
|  | Internet time | 0.10 | 2.61** |  | -0.03 | -0.93 |  | 0.05 | 1.19 |
|  | Extraversion | -0.06 | -1.53 |  | -0.31 | -8.45** |  | 0.16 | 3.91** |
|  | *Self-Identity Integration* | -0.19 | -4.66** |  | -0.23 | -5.99** |  | 0.03 | 0.59 |
|  | *Relationship Integration* | -0.05 | -1.13 |  | -0.10 | -2.67** |  | 0.10 | 2.21* |
|  | *Social Function Integration* | -0.25 | -6.48** |  | -0.13 | -3.53** |  | 0.17 | 4.17** |

^a^ Gender was coded as male = 1, female = 0.

^b^ Internet time was measured as the number of online hours per day.

* *p*<0.05, ** *p*<0.01.
